# Association of truncal involvement in lupus erythematosus panniculitis patients with calcinosis cutis: A retrospective cohort study

**DOI:** 10.1177/09612033251349451

**Published:** 2025-06-08

**Authors:** Jay Garza, Benjamin F. Chong

**Affiliations:** Department of Dermatology, 12334 University of Texas Southwestern Medical Center, Dallas, TX, USA

**Keywords:** Cutaneous lupus erythematosus, lupus erythematosus panniculitis, calcinosis cutis, discoid lupus

## Abstract

**Background:**

Lupus erythematosus panniculitis (LEP) is a rare variant of cutaneous lupus erythematosus that typically presents as indurated nodules or plaques. Calcinosis cutis (CC) is a potential complication of this disease with limited treatment modalities and significant quality of life complications. The rate and risk factors of CC in LEP are not well understood. Thus, we conducted a retrospective cohort study on patients diagnosed with LEP.

**Objective:**

To quantify the rate of CC and to identify the risk factors associated with its development in LEP.

**Methods:**

This retrospective cohort study analyzed data from 27 LEP patients recruited in outpatient dermatology clinics at University of Texas Southwestern Medical Center and Parkland Health from January 2009 to December 2024. The primary outcome measure was CC development based on clinical diagnosis from a dermatologist, biopsy, or radiographic imaging. Data collected included demographics, smoking history, disease duration, medications, and lesion location. Predictor variables associated with CC development were analyzed either by Mann-Whitney U or Fisher’s exact tests.

**Results:**

10/27 (37%) LEP patients had CC during the evaluation period. LEP patients with CC had a higher rate of truncal involvement (9/10 (90%) versus 7/17 (41.2%); *p* = .02) and a lower rate of head & neck involvement (3/10 (30%) versus 13/17 (76.5%); *p* = .04) of their LEP lesions compared to those without CC.

**Limitations:**

This study is limited by its single-center design, retrospective nature, and small sample size.

**Conclusions:**

This cohort of LEP patients had over a third developing CC. LEP lesion location significantly differed in those who developed CC compared with those who did not. CC is a common complication of LEP that requires close monitoring by clinicians. Prospective multicenter studies are needed to confirm these findings and better understand the predictive factors for the development of CC in LEP patients.

## Introduction

Lupus erythematosus panniculitis (LEP) is a subtype of cutaneous lupus erythematosus (CLE) that can present as tender subcutaneous nodules that evolve into indurated and atrophic plaques. LEP lesions can be solitary or they can involve multiple areas.^1–4^ LEP can often cause morbidity with bothersome symptoms such as pain and leaving significant irreversible skin damage such as skin atrophy.^1,3^ The range of age of onset has been reported to fall between 30 and 60 years old with the apex between the mid-30s or early 40s.^1,2^

A problematic sequela of LEP is calcinosis cutis,^1–3^ which is the deposition of calcium salts in the dermis and subcutaneous fat that can cause skin discomfort. CC can typically be observed and diagnosed clinically; however, imaging can also confirm the diagnosis with plain radiography as the first-line modality.^5^ While there are five subtypes of CC, LEP primarily causes dystrophic CC, whose treatment options including calcium channel blockers, are limited by insufficient efficacy.^1,5^ Several mechanisms have been proposed for development of CC in autoimmune cutaneous diseases including chronic inflammation, vascular hypoxia, and recurrent trauma.^5–8^

While the pathophysiology for the development of CC in LEP has yet to be elucidated, chronic inflammation has been suggested to play a role.^1,4^ Similarly, CC appears to be the result of chronic inflammation in juvenile and adult dermatomyositis.^5,7,9^ Chronic inflammation can result in cell necrosis, where denatured proteins released from necrotic cells can bind readily to phosphate ions and subsequently attach to calcium, acting as a nidus for calcification.^6^ Another contributing component of dystrophic CC involves abnormally high mitochondrial calcium phosphate levels resulting in crystallization and cell death.^6^ There is also in vitro evidence that elevated levels of pro-inflammatory cytokines, including interleukin (IL)-1β, IL-6, and tumor necrosis factor (TNF)-α, in juvenile dermatomyositis can lead to subcutaneous and cutaneous calcification.^5,7^ These cytokines are thought to play a role in calcification through enhanced expression of RUNX2 (runt-related transcription factor 2) which facilitates osteoblast differentiation.^7^ Other reported mechanisms for the development of dystrophic CC include trauma and vascular compromise, namely for systemic sclerosis.^5,8^ History of frequent digital ulceration and elevated levels of hypoxia-associated glucose transporter 1 molecule supports the roles of trauma and vascular compromise in fostering calcinosis cutis lesions within patients with systemic sclerosis.^5,8,10^

Studies have previously reported rates of CC in LEP ranging from 10% to 34%, and the most common areas of LEP with CC include upper limbs, face, buttocks, and lower limbs.^2,3,11^ However, these studies have relied solely on the histopathologic diagnosis of calcinosis cutis and/or utilization of international classification of diseases (ICD) codes. To address these prior limitations, we intend to identify CC in our LEP patient cohort through histopathologic, clinical, and/or radiographic data and confirm the diagnosis by a dermato-rheumatologic expert physician. We also utilized clinical photography to identify body location involvement. This report aims to quantify the rate of development of CC and to determine the risk factors associated with its development in a diverse cohort of LEP patients.^2,3^

## Methods

### Participants

We conducted a retrospective cohort study examining patients with CLE enrolled at the University of Texas Southwestern (UTSW) Cutaneous Lupus Registry at outpatient dermatology clinics at UTSW Medical Center or Parkland Health from 1 January 2009, to 31 December 2024. Patients who were above the age of 18 and had a diagnosis of LEP were included. We excluded those with insufficient clinical data. LEP and CC diagnoses were confirmed by a dermatologist (B.F.C.) using clinicopathological correlation. Systemic lupus erythematosus (SLE) diagnosis was made using ACR 1997 criteria. All patients offered informed consent, and the University of Texas Southwestern Internal Review Board had given its approval.

### Data collection

Patient demographic and clinical information such as anti-nuclear antibody (ANA) laboratory values, smoking history, and treatment history were collected through questionnaires and electronic medical record review. Age of the patient and date of LEP and/or CC diagnosis were determined on the day that the patient first received their diagnosis from a dermatologist. Race and ethnicity were self-reported. Racial groups initially included White, Black, Asian, Native American/Alaskan Native, and other/mixed races. Lesion location was identified retrospectively via photography and physical exam correlation.

### Statistical analysis

Univariate analyses including Fisher’s exact test or Mann-Whitney U tests were performed on categorical and continuous variables collected, respectively. Analyses were performed using GraphPad Prism 10. All statistical tests were two-sided and *p*-values < .05 were considered significant.

## Results

27 patients with LEP were included in this retrospective cohort study. 20 (74.1%) were female, and 18 (66.7%) were Black, non-Hispanic. The other racial/ethnic demographic representations were three (11.1%) Asian non-Hispanic, three White Hispanic, and three White non-Hispanic. The median age of diagnosis of LEP was 34.5 years old (interquartile range (IQR): 24.5–41). The median follow-up duration for all LEP patients was 4.8 years (IQR: 2.5–10.6). The median duration from rash onset to the date of diagnosis of LEP was 18.0 months (IQR: 5.3–27.8) (Table 1). 25 (92.6%) out of the 27 had shown LEP activity during their follow-up period.

**Table 1.** Univariate analyses of demographic and clinical characteristics.

	All LEP patients (*N* = 27)	LEP w/ CC (*N* = 10)	LEP w/o CC (*N* = 17)	*p*-value
Age at LEP diagnosis (years) (median, IQR)	34.5 (24.5–41)	38.5 (32.3–47.5)	33 (23–37)	*p* = .14
Gender (*N*, %)				
Male	7 (25.9%)	2 (20%)	5 (29.4%)	*p* = .68
Female	20 (74.1%)	8 (80%)	12 (70.6%)	
Race/Ethnicity (*N*, %)				
Black, non-Hispanic	18 (66.7%)	7 (70%)	11 (64.7%)	*p* > .99
Other	9 (33.3%)	3 (30%)	6 (35.3%)	
LEP to CC diagnosis (years)^a^ (median, IQR)	N/A	5.1 (0.3–6.9)	N/A	
Follow-up duration (years)^b^ (median, IQR)	4.8 (2.5–10.6)	7.0 (3.2–11.0)	4.0 (2.4–9.5)	*p* = .60
Rash onset to Dx (months)^c^ (median, IQR)	18.0 (5.3–27.8)	18.0 (12.6–23.5)	12.3 (5.1–29.7)	*p* = .81
Smoking history (ever) (*N*, %)	10 (37%)	5 (50%)	5 (29.4%)	*p* = .42
CLASI-A (median, IQR)	2 (0-4)	3 (0.5–6.25)	1 (0–4)	*p* = .29
Medication history (*N*, %)				
Topicals + Antimalarials	14 (51.9%)	4 (40%)	10 (58.8%)	*p* = .44
Topicals + Antimalarials + other immunosuppressants^d^	13 (48.1%)	6 (60%)	7 (41.2%)	
Concurrent diagnoses (*N*, %)				
DLE	19 (70.4%)	7/10 (70%)	12/17 (71%)	*p* > .99
SLE	13 (48.1%)	6/10 (60%)	7/17 (41.2%)	*p* = .44
Malar rash	2 (15.3%)	1 (16.7%)	1 (14.3%)	*p* > .99
Discoid rash	9 (69.2%)	5 (83.3%)	4 (57.1%)	*p* = .23
Photosensitivity	11 (84.6%)	4 (66.7%)	7 (100%)	*p* > .99
Oral ulcers	1 (7.7%)	1 (16.7%)	0	*p* = .37
Arthritis	5 (38.5%)	3 (50%)	2 (28.6%)	*p* = .33
Renal disease	2 (15.4%)	1 (16.7%)	1 (14.3%)	*p* > .99
Hematologic Disorder	9 (69.2%)	4 (66.7%)	5 (71.4%)	*p* = .68
Immunologic Disorder	11 (84.6%)	5 (83.3%)	6 (85.7%)	*p* = .69
ANA (+)	13 (100%)	6 (100%)	7 (100%)	*p* = .44
Positive ANA (≥1:160)	24 (88.9%)	10 (100%)	14 (82.4%)	*p* = .27

Abbreviations: CC: calcinosis cutis; DLE: discoid lupus erythematosus; LEP: lupus erythematous panniculitis; SLE: systemic lupus erythematosus.

^a^LEP diagnosis date to CC diagnosis date.

^b^LEP diagnosis date to last follow-up visit.

^c^Patient-reported rash onset to LEP diagnosis were available in 7 LEP patients with CC and 14 LEP patients without CC.

^d^Immunosuppressants ever taken in the LEP with CC group included mycophenolate mofetil (*N* = 5), methotrexate (*N* = 4), azathioprine (*N* = 3), belimumab (*N* = 2), leflunomide (*N* = 1), and intravenous immunoglobulin (*N* = 1). Immunosuppressants ever taken in the LEP without CC group included mycophenolate mofetil (*N* = 6), thalidomide (*N* = 2) methotrexate (*N* = 1), azathioprine (*N* = 1), rituximab (*N* = 1), lenalidomide (*N* = 1).

**Figure 1.**
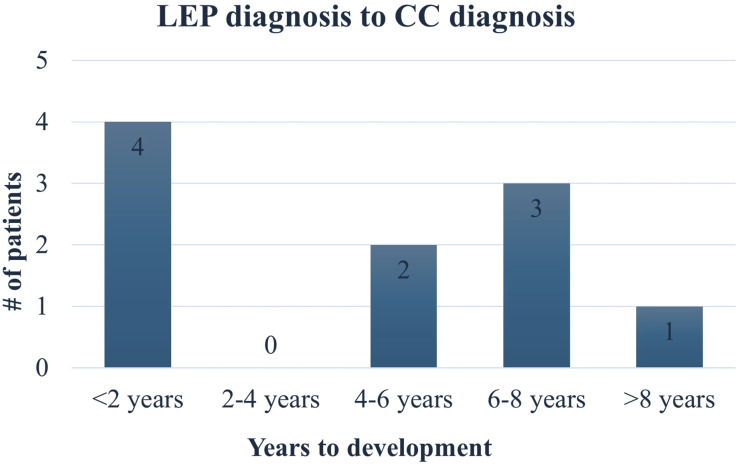
Time from diagnosis of lupus erythematosus panniculitis (LEP) to diagnosis of calcinosis cutis (CC). This bar graph shows the frequency and range of time between diagnosis of LEP to the diagnosis of CC splitting patients into groups of 2-year time slots. This illustrates that the majority of patients developed CC 4 years after diagnosis of LEP.

Of the 27 LEP patients, 10 (37%) had the presence of CC. Of the 10 LEP patients that developed CC, there were eight (80%) females. Seven (70%) were Black, non-Hispanic, and there was one Asian non-Hispanic, one White Hispanic, and one White non-Hispanic. The median duration from diagnosis of LEP to diagnosis of CC was 5.1 years (IQR 0.3–6.9) (Table 1). Four LEP patients were diagnosed with CC within 2 years of their diagnosis with LEP. The other six were diagnosed with CC more than 4 years after their LEP diagnosis, ranging from 5.0 years to 11.6 years (Figure 1).

Univariate analyses were performed to identify patient factors associated with CC development in LEP. LEP patients with CC were statistically more likely to have truncal involvement (90% vs 41.2%, *p* = .02) and less likely to have head & neck involvement of their LEP lesions (30% v. 76.5%, *p* = .04) compared to their counterparts without CC (Table 2). The LEP lesions that contained CC were located in all body site groups but were predominantly found in the trunk (6 patients) and proximal upper extremities (5 patients). Other patient factors including smoking history, medication history, concurrent lupus diagnoses (DLE, SLE), or ANA positivity were not found to be significantly different between the two groups (Table 1).

**Table 2.** Frequency of lupus erythematosus panniculitis (LEP) lesions by body site in LEP patients with and without calcinosis cutis (CC).

Body site^a^	LEP w/CC (*N* = 10)^b^	LEP w/o CC (*N* = 17)	
Head & neck^c^	3 (30%)	13 (76.5%)	*p* = .04
Scalp	0	4 (23.5%)	
Malar area	0	5 (29.4%)	
Rest of face	3 (30%)	9 (53%)	
Trunk^c^	9 (90%)	7 (41.2%)	*p* = .02
Chest	4 (40%)	1 (5.9%)	
Abdomen	2 (20%)	0	
Buttocks	5 (50%)	3 (17.6%)	
Back	3 (30%)	5 (29.4%)	
Upper extremities	5 (50%)	8 (47.1%)	*p* > .99
Lower extremities	5 (50%)	4 (23.5%)	*p* = .22

Abbreviations: CC: calcinosis cutis, LEP: lupus erythematosus panniculitis.

^a^Each patient may have at least one LEP lesion per body site.

^b^CC was found on the trunk of 6 patients (3 chest, 1 abdomen, 4 buttocks, 1 back), on the arms of 5 patients, on the legs of 2 patients, and on the face of 1 patient.

^c^Patients with LEP lesions on head and neck or trunk with more than one sub-area of involvement were counted once.

## Discussion

In this retrospective cohort study, we reported over one-third, namely 37%, of LEP patients developing CC. The median duration of LEP to onset of CC was about 5 years. LEP patients with CC also had a preponderance of truncal involvement of their LEP lesions but fewer head and neck lesions compared to their counterparts without CC. CC lesions were most commonly found on the trunk and arms.

The frequency of CC found in our LEP cohort (37%) is in the upper range of previously reported rates.^2,3,11^ Arai et al and Sanchez et al, which reported 10% and 34% in their cohorts, respectively, both relied on histological diagnosis of CC.^3,11^ Diagnosis of CC solely via histology may present a lower rate due to sampling bias. On the contrary, the detection methods for CC utilized in our group was distributed with five diagnoses made radiographically, two via clinician diagnosis, and three by biopsy. Our report suggests that CC can be a common complication associated with LEP, and potentially similar in frequency to dermatomyositis and systemic sclerosis.^5,9,10,12,13^ The frequency of calcinosis cutis in systemic sclerosis has been reported in the range of 18–25%.^10,13^

The LEP patients that developed CC showed a propensity towards having truncal LEP lesions. Although these patients’ truncal LEP lesions did not always develop CC, these patients had a higher rate of LEP lesions with CC elsewhere on their body. We also found that the LEP patients with CC were less likely to have head and neck involvement of their LEP lesions. These findings suggest that the involvement of certain body sites predispose patients to the development of CC. Thus, clinicians can take this helpful information in performing full body examinations in LEP patients, noting that those with truncal lesions could be at higher risk of having CC.

We found that the median duration of LEP diagnosis to the onset of CC was 5.1 years. This was similar to Balin et al., who reported an average time to onset of 4.8 years for four LEP patients.^12^ In contrast, our reported LEP duration of disease to onset of CC was shorter than what has been reported for systemic sclerosis (7.5–9.4 years) and adult dermatomyositis patients (∼8 years).^10,12^ Longer duration of LEP has been suggested to increase predilection for CC,^1,4^ but we were unable to confirm this hypothesis in our study. We did not find that any indicators of LEP chronicity such as duration of LEP disease, time to LEP onset, and follow up duration to be significantly different in LEP patients with CC versus those without CC. Since chronic inflammation may play a role in CC development, an association between duration of LEP lesions and CC is still possible but needs to be further explored with larger cohort studies.

Additionally, we showed that there were no statistically significant differences in other demographics including age, sex, race, and smoking history between those with LEP that had developed CC and that did not. This may be due to the limited sample size in this study. Smoking cessation has been suggested in previous literature to play a role in management of CC in rheumatic diseases including the prevention of developing new CC nodules and mitigation of symptoms from CC.^5^ However, other reports show there is no association with positive smoking history and development of calcinosis cutis in systemic sclerosis.^10,13^ Larger cohort studies are warranted to determine the role smoking plays in CC development in LEP.

Notably, the frequency of CC in systemic lupus erythematosus (SLE) is low.^12^ The few cases in literature are also found to have mild symptoms.^6,12^ Balin et al. also reported that the time to onset of CC in four SLE patients (mean: 258 months (range: 228–288 months)) was longer compared to two patients with LEP (mean: 58 months (range: 5–108 months).^12^ Rothe et al. described three cases of SLE complicated by CC occurring in upper and lower extremities and buttocks and included 23 other similar cases previously reported in literature.^14^ To further elaborate on their findings, three patients of the total 26 had overlying discoid lesions in the same area as their calcinosis cutis. Also, subcutaneous fat necrosis was observed in three patients; however, they did not have features suggestive of LEP. In summary, CC in SLE typically affects the extremities and buttocks and can take longer time to development compared to other rheumatic diseases such as scleroderma and dermatomyositis.^6,12,14^

Overall, our cohort showed that over one third of LEP patients had developed CC over a median period of a little more than 5 years. These patients showed a higher rate of LEP truncal involvement and a lower rate of head and neck LEP involvement. These findings suggest that vigilant monitoring of LEP lesions on patients with truncal involvement is warranted. Our study was limited by its small sample size, single center design, and retrospective analysis. Serological studies were also limited to ANA for our patients, as other autoantibodies were not available or ordered in most LEP patients. Future larger prospective multicenter studies will be carried out to confirm our findings and further delineate the risk factors associated with the development of CC in LEP. While there are no consistently effective treatments for CC, early detection and prompt initiation of the appropriate pharmaceuticals such as calcium channel blockers and anti-inflammatory agents may decrease morbidity for patients.

## Appendix

### Abbreviations

ANA: Antinuclear antibody
CC: Calcinosis cutis
DLE: Discoid lupus erythematosus
IQR: Interquartile range
LEP: Lupus erythematosus panniculitis
SLE: Systemic lupus erythematosus
UTSW: University of Texas Southwestern
